# Impact of fasting duration on LDL cholesterol concentrations estimated by the Friedewald, Martin-Hopkins, and Sampson/NIH equations

**DOI:** 10.11613/BM.2025.020704

**Published:** 2025-04-15

**Authors:** Şerif Ercan, Ali Manav

**Affiliations:** 1 Department of Medical Biochemistry, Lüleburgaz State Hospital, Kırklareli, Türkiye; 2 Department of Cardiology, Lüleburgaz State Hospital, Kırklareli, Türkiye

**Keywords:** cholesterol, fasting, lipids, postprandial lipemia, preanalytical phase

## Abstract

**Introduction:**

A paradigm shift is occurring in lipid testing, as fasting is no longer required. We aimed to determine whether low-density lipoprotein cholesterol (LDL-C) concentrations calculated using three different equations, along with the components used in these calculations, vary with different fasting durations in routine clinical practice.

**Materials and methods:**

The concentrations of LDL-C were calculated using the Friedewald, Martin-Hopkins, and Sampson/NIH equations, along with the lipid components involved in these equations, depending on time since the last meal in a cohort of 77,300 outpatients at a community hospital. The study population was divided into groups according to fasting durations by 2-hour intervals. A general linear model was applied to identify differences between fasting and nonfasting groups.

**Results:**

Regardless of the calculation method, LDL-C concentrations varied with fasting duration for up to 8-10 hours. The greatest absolute mean differences in LDL-C concentrations between fasting and nonfasting states were - 0.32, - 0.30, and - 0.26 mmol/L when using the Friedewald, Sampson/NIH, and Martin-Hopkins equations, respectively. Among the equation components, triglyceride concentrations were the most sensitive to fasting duration, remaining elevated for 4-6 hours after the last meal, while total cholesterol and non-high-density lipoprotein cholesterol (HDL-C) concentrations decreased for up to 8-10 hours postprandially. However, HDL-C concentrations remained relatively stable.

**Conclusions:**

The variation in postprandial LDL-C concentrations was observed not to differ between the three calculation methods and reached negligible concentrations after at least 8 hours of fasting. If LDL-C concentrations measured in a nonfasting state are near clinical decision thresholds, subsequent lipid measurement should be performed in a fasting state.

## Graphical abstract

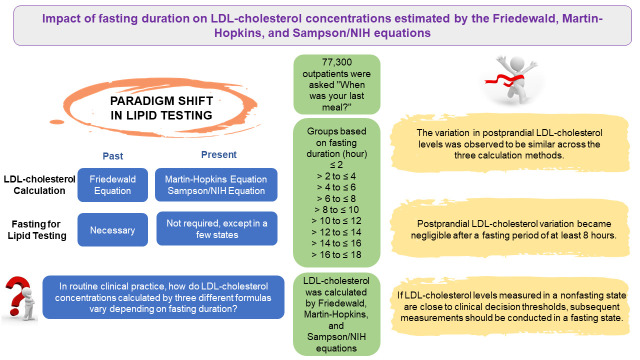

## Highlights

• Low-density lipoprotein cholesterol (LDL-C) has been frequently calculated using the Friedewald equation for several decades

• The guidelines today recommend the Martin-Hopkins and Sampson/NIH formulas

• There is a paradigm shift in lipid testing currently: fasting is no longer required

• Postprandial LDL-C variation was similar across the three calculation methods

• Postprandial LDL-C variation was negligible after at least 8 hours of fasting

## Introduction

Low-density lipoprotein cholesterol (LDL-C) is recognized as a primary contributor to atherosclerotic cardiovascular disease (*1*-*4*). Recent guidelines from both the European Society of Cardiology/European Atherosclerosis Society (ESC/EAS) and the American College of Cardiology/American Heart Association (ACC/AHA) emphasize the crucial role of LDL-C in cardiovascular risk stratification and in assessing the effectiveness of lipid-lowering treatments (*1*, *4*). Consequently, accurate LDL-C determination is essential for appropriate clinical interventions.

Measurement of LDL-C can be performed using the beta-quantification procedure, which combines ultracentrifugation and precipitation techniques, or through direct homogeneous methods (*5*). However, for several decades, LDL-C has been estimated from a standard lipid profile that includes measurements of total cholesterol, high-density lipoprotein cholesterol (HDL-C), and triglycerides using an equation proposed by James Friedewald in 1972 (*6*). The equation assumes a constant ratio between triglyceride and very-low-density lipoprotein cholesterol (VLDL-C) under fasting conditions. However, the use of a fixed coefficient to estimate VLDL-C becomes problematic when the serum triglyceride concentration exceeds 2.3 mmol/L (200 mg/dL) and is invalid beyond 4.5 mmol/L (400 mg/dL) (*7*, *8*). The same issue occurs at lower LDL-C concentrations (*9*).

In clinical practice, high triglyceride and/or low LDL-C are becoming more common due to the emergence of new and potent LDL-C-lowering medications (*3*). Additionally, the increasing incidence of obesity, metabolic syndrome, and diabetes mellitus has contributed to the rising prevalence of hypertriglyceridemia (*5*).

Moreover, contemporary practice has seen a notable paradigm shift concerning lipid testing, where fasting is no longer routinely required before blood sampling (*1*-*4*, *10*). Nonfasting samples may contain chylomicrons, which can have a higher triglyceride content than VLDL particles, depending on the fat content of a meal and the time since consumption (*7*). This can result in an erroneous overestimation of VLDL-C and consequently an underestimation of LDL-C values calculated by the Friedewald equation.

Several equations have been proposed to address these issues in LDL-C calculation. The Martin-Hopkins equation, developed by Martin *et al.* in 2013, is similar to the Friedewald equation but uses an individualized factor based on triglyceride and non-HDL-C values to estimate VLDL-C by dividing triglycerides, instead of a fixed factor (*11*). This method is recommended by the consensus paper of EAS/European Federation of Clinical Chemistry and Laboratory Medicine (EFLM) for determining LDL-C in cases of moderately elevated triglyceride and/or low LDL-C concentrations, as well as by the ACC/AHA guideline (*2*, *4*). It is also suggested that the Martin-Hopkins equation might be preferable for calculating LDL-C in a nonfasting state (*2*, *4*).

In 2020, Sampson *et al.* proposed another equation, known as the Sampson/National Institutes of Health (NIH) equation, for LDL-C estimation where VLDL-C was estimated using multiple least squares regression (*12*). This estimation of LDL-C is claimed to be highly accurate up to 9.04 mmol/L (800 mg/dL) of triglycerides (*12*). The Canadian Society of Clinical Chemists recommends adopting the Sampson/NIH equation for calculating LDL-C in all patients, rather than using the Friedewald equation (*13*).

Previous studies have often focused on the accuracy of the new LDL-C calculation equations by comparing the estimated LDL-C results with those obtained from reference methods or direct homogeneous methods (*8*, *14*). However, there is no data available regarding the influence of fasting status on the two new approaches. Therefore, this study aimed to examine the impact of different fasting durations on the LDL-C concentrations calculated using the Friedwald, Martin-Hopkins, and Sampson/NIH equations, as well as the components used in these calculations.

## Material and methods

### Subjects

This cross-sectional study was conducted using data from the laboratory information system covering the period from April 2021 to June 2023 at Lüleburgaz State Hospital, Kırklareli, Türkiye. A total of 77,300 outpatients, who were requested by their physicians to undergo blood collection for a standard lipid profile, were enrolled in this study. Participants ranged in age from 18 to 101 years.

This study was approved by the Kırklareli University Institute of Health Sciences Ethics Committee (Approval Number: 15.3.2021/5) and was conducted according to the Declaration of Helsinki.

### Methods

In early 2020, our laboratory ceased the requirement for fasting in lipid tests. In line with this change, information about the time of the last meal was collected from all patients presenting to the blood collection sites for lipid measurement, and this data was recorded in the laboratory information system.

The fasting duration was calculated for each individual as the difference, in hours, between the time of blood sampling and the time since their last meal. Participants with an unknown time since their last meal were excluded from the analysis.

The study population was divided into nine groups based on fasting duration, with 2-hour intervals of ≤ 2, > 2 to ≤ 4, > 4 to ≤ 6, > 6 to ≤ 8, > 8 to ≤ 10, > 10 to ≤ 12, > 12 to ≤ 14, > 14 to ≤ 16, and > 16 to ≤ 18 hours.

Blood samples were collected into serum tubes with a gel separator (8 mL, VACUETTE Z Serum Sep Clot Activator, Greiner Bio-One, Kremsmünster, Austria). All serum tubes were allowed to clot for 30 minutes at room temperature and then centrifuged at 2000xg for 10 minutes.

Enzymatic methods were used to measure serum concentrations of triglycerides, total cholesterol, and HDL-C on the Cobas 6000 c501 analyzers (Roche Diagnostic GmbH, Mannheim, Germany), with HDL-C assessed after the combination of non-HDL lipoproteins with polyanions and a detergent.

All measurements were monitored for precision through daily assessments using two-level internal quality control (IQC) samples (PreciControl ClinChem Multi 1 and 2) provided by the analyzer manufacturer (Roche Diagnostics, Mannheim, Germany). During the study period, two different lot-numbered IQC samples were used for each IQC level. The mean concentrations of IQC Level 1 samples were 1.29 and 1.33 mmol/L for triglycerides, 2.29 and 2.82 mmol/L for total cholesterol, and 0.8 and 0.87 mmol/L for HDL-C, while those of IQC Level 2 samples were 2.34 and 2.46 mmol/L for triglycerides, 4.29 and 4.4 mmol/L for total cholesterol, and 1.44 and 1.68 mmol/L for HDL-C.

For accuracy, monthly evaluations were conducted using external quality control (EQC) samples obtained from the Randox International Quality Assessment Scheme (Randox Laboratories Ltd., Dublin, United Kingdom).

Estimation of LDL-C was performed separately using the Friedewald, Martin-Hopkins, and Sampson/NIH equations.

The calculation for LDL-C *via* the Friedewald equation was performed using Eq. 1:



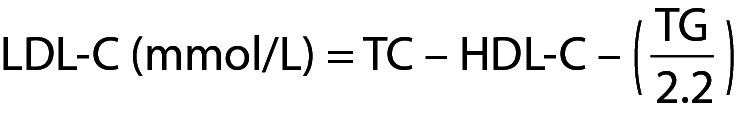



where TC is the total cholesterol concentration (*6*).

Calculation of LDL-C using the Martin-Hopkins equation was performed as follows (Eq. 2):



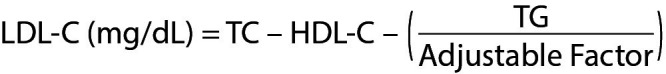



where TC is the total cholesterol concentration (*10*). The adjustable factor, ranging from 3.1 to 9.5, was previously presented in a 180-cell table based on triglyceride and non-HDL-C concentrations (*12*).

Non-HDL-C was calculated as total cholesterol minus HDL-C. Concentrations of LDL-C were converted from mg/dL to mmol/L by multiplying with a factor of 0.0259.

The LDL-C calculated using the Sampson/NIH equation is as follows (Eq. 3):



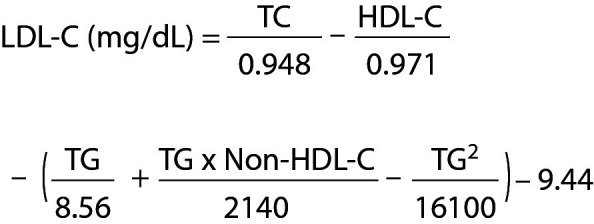



where TC is total cholesterol concentration, and TG is triglyceride concentrations (*12*). Conversion of LDL-C concentrations from mg/dL to mmol/L was performed by multiplying with a factor of 0.0259.

Despite the claim that the Sampson equation is valid up to 9.03 mmol/L (800 mg/dL), LDL-C concentrations were not calculated for subjects with triglyceride concentrations greater than 4.52 mmol/L (400 mg/dL) because the Friedewald and Martin-Hopkins equations are specifically validated for triglyceride concentrations below 4.52 mmol/L (*6*, *11*).

### Statistical analysis

The baseline lipid characteristics, calculated using data from all included participants, are presented as mean ± standard deviation.

A general linear model was conducted to examine whether there were differences in the means of groups based on time since the last meal for LDL-C estimates and lipid measurements.

In addition to fasting duration, age, and gender were included in the model as fixed factors, while blood collection time was included as a covariate. For this purpose, the age range from 18 to 78 years was categorized into groups at 5-year intervals, with individuals older than 78 years grouped into a final category. If the investigated analyte varied as a function of the factors and their interactions, estimated marginal means with 95% confidence intervals were calculated for each group based on fasting durations. Accordingly, the data are presented as estimated marginal means ± standard error of the mean.

The Bonferroni *post hoc* test was used for multiple pairwise comparisons. A significant heterogeneity has been reported in the definitions of fasting used by healthcare workers and in the literature (*15*). Following the EFLM paper by the Working Group for Preanalytical Phase (WG-PRE), which defines a fasting state as 12 hours after the last meal, we set the reference fasting duration at 12 to 14 hours (*16*).

We also calculated the mean difference, both in absolute terms and as percentages, between LDL-C estimates and lipid measurements obtained after a fasting duration of 12 to 14 hours and those obtained from other fasting time intervals. These differences were then compared with the biological variation of the analyte of interest, as cited in the EFLM Biological Variation Database (*17*). The within-subject biological variation values were 7.7%, 5.2%, 5.7%, and 19.7% for LDL-C, total cholesterol, HDL-C, and triglyceride, respectively (*17*).

Statistical analyses were conducted using SPSS Statistics version 21 (IBM, New York, USA). Statistical significance was defined as P < 0.05.

## Results

After excluding 3737 individuals due to missing fasting times and an additional 2189 individuals due to triglyceride concentrations exceeding 4.52 mmol/L, 71,374 subjects were included in the analyses.

Baseline characteristics of the study population are shown in Table 1. The participants had a median age of 54 years (range 18-101), and 63% were female. Blood samples were collected in the morning for 94% of individuals (before 12:00 h).

**Table 1:** Baseline characteristics of study population

**Characteristic**	**Study population** **(N = 71,374)**
Age (year), median (range)	54 (18-101)
**Age category (year)**	**N (%)**
> 18 to ≤ 23	3829 (5.4%)
> 23 to ≤ 28	2854 (4.0%)
> 28 to ≤ 33	3078 (4.3%)
> 33 to ≤ 38	3926 (5.5%)
> 38 to ≤ 43	5494 (7.7%)
> 43 to ≤ 48	6856 (9.6%)
> 48 to ≤ 53	8189 (11.5%)
> 53 to ≤ 58	8687 (12.2%)
> 58 to ≤ 63	8932 (12.5%)
> 63 to ≤ 68	8070 (11.3%)
> 68 to ≤ 73	5655 (7.9%)
> 73 to ≤ 78	3220 (4.5%)
> 78	2584 (3.6%)
**Gender**	**N (%)**
Female	45,219 (63.4%)
Male	26,155 (36.6%)
**Blood collection time (hour)**	**N (%)**
≥ 08:00 to < 10:00	34,527 (48.4%)
≥ 10:00 to < 12:00	32,307 (45.3%)
≥ 12:00 to < 14:00	2697 (3.8%)
≥ 14:00 to < 16:00	1725 (2.4%)
≥ 16:00 to < 17:00	118 (0.2%)
**Lipid values**	**mean ± SD (mmol/L)**
LDL-C by Friedewald equation	3.10 ± 1.01
LDL-C by Martin-Hopkins equation	3.19 ± 1.00
LDL-C by Sampson/NIH equation	3.18 ± 1.00
Triglyceride	1.69 ± 0.82
Total cholesterol	5.18 ± 1.17
HDL-C	1.30 ± 0.34
Non-HDL-C	3.88 ± 1.15
LDL-C - low density lipoprotein cholesterol. HDL-C - high density lipoprotein cholesterol. SD - standard deviation.	

During the 27-month study period, the monthly CV values for triglycerides at the IQC level 1 ranged from 0.54% to 4.57%, with an average of 1.68%. Similarly, for level 2, the CV ranged from 0.22% to 4.34%, with an average of 1.48%. The CV values for total cholesterol at IQC level 1 ranged from 0.47% to 3.68%, and for IQC level 2, the range was from 0.78% to 3.55%, with respective averages of 1.76% and 1.71%. Lastly, the CV values for HDL-C ranged from 0.78% to 3.83% for level 1 and from 0.71% to 3.17% for level 2, with corresponding averages of 1.74% and 1.69%, respectively.

Additionally, during this study period, the standard deviation index values calculated from the EQC results for each month were consistently ranged between - 2 and 2 for all three tests, with ranges of 0 to 1.75 for triglycerides, - 1.96 to 0.03 for total cholesterol, and 0 to 0.51 for HDL-C.

Table 2 presents the estimated marginal mean concentrations of the lipid parameters for each fasting state, along with absolute and percentage mean differences between fasting for > 12 to ≤ 14 hours and the other states.

**Table 2:** Estimated marginal mean values of lipid parameters based on time since the last meal, with mean differences between the fasting state of >12 to ≤14 hours and other fasting durations

**Time since last meal**	**N**	**Estimated marginal mean (95% CI)**	**Standard error (SE)**	**Absolute mean difference (95% CI)**	**Percent mean difference (%)**	**P**
**LDL-C by Friedewald, mmol/L**						
≤ 2	573	2.69 (2.6 to 2.78)	0.04	- 0.32 (- 0.47 to - 0.17)	- 10.6	< 0.001
> 2 to ≤ 4	1272	2.7 (2.64 to 2.76)	0.03	- 0.31 (- 0.42 to - 0.2)	- 10.3	< 0.001
> 4 to ≤ 6	979	2.73 (2.66 to 2.81)	0.04	- 0.28 (- 0.41 to - 0.15)	- 9.3	< 0.001
> 6 to ≤ 8	564	2.82 (2.73 to 2.91)	0.05	- 0.19 (- 0.34 to - 0.04)	- 6.3	0.002
> 8 to ≤ 10	1238	3.01 (2.94 to 3.07)	0.03	0 (- 0.11 to 0.11)	0.0	1.000
> 10 to ≤ 12	3172	2.97 (2.93 to 3.01)	0.02	- 0.04 (-0.11 to 0.03)	- 1.3	1.000
> 12 to ≤ 14	31,629	3.01 (3 to 3.02)	0.01			
> 14 to ≤ 16	28,740	3.02 (3 to 3.03)	0.01	0.01 (- 0.03 to 0.04)	0.3	1.000
> 16 to ≤ 18	3207	2.97 (2.93 to 3.01)	0.02	- 0.04 (- 0.12 to 0.04)	- 1.3	1.000
≤ 2	573	2.83 (2.74 to 2.91)	0.04	- 0.26 (- 0.41 to - 0.12)	- 8.4	< 0.001
> 2 to ≤ 4	1272	2.84 (2.77 to 2.9)	0.03	- 0.25 (- 0.36 to - 0.15)	- 8.1	< 0.001
**LDL-C by Martin-Hopkins, mmol/L**						
> 4 to ≤ 6	979	2.86 (2.79 to 2.94)	0.04	- 0.23 (- 0.35 to - 0.1)	- 7.4	< 0.001
> 6 to ≤ 8	564	2.92 (2.83 to 3.01)	0.04	- 0.17 (- 0.32 to - 0.02)	- 5.5	0.007
> 8 to ≤ 10	1238	3.08 (3.02 to 3.15)	0.03	- 0.01 (- 0.11 to 0.1)	- 0.3	1.000
> 10 to ≤ 12	3172	3.05 (3.01 to 3.1)	0.02	- 0.04 (- 0.11 to 0.03)	- 1.3	1.000
> 12 to ≤ 14	31,629	3.09 (3.08 to 3.11)	0.01			
> 14 to ≤ 16	28,740	3.09 (3.08 to 3.11)	0.01	0 (- 0.03 to 0.04)	0.0	1.000
> 16 to ≤ 18	3207	3.05 (3.01 to 3.09)	0.02	- 0.04 (- 0.11 to 0.03)	- 1.3	1.000
≤ 2	573	2.78 (2.7 to 2.87)	0.04	- 0.3 (- 0.45 to - 0.16)	- 9.7	< 0.001
> 2 to ≤ 4	1272	2.79 (2.73 to 2.85)	0.03	- 0.29 (- 0.4 to - 0.18)	- 9.4	< 0.001
> 4 to ≤ 6	979	2.82 (2.75 to 2.9)	0.04	- 0.26 (- 0.39 to - 0.13)	- 8.4	< 0.001
**LDL-C by Sampson/NIH, mmol/L**						
> 6 to ≤ 8	564	2.9 (2.82 to 2.99)	0.04	- 0.18 (- 0.33 to - 0.03)	- 5.8	0.003
> 8 to ≤ 10	1238	3.08 (3.01 to 3.14)	0.03	0 (- 0.11 to 0.1)	0.0	1.000
> 10 to ≤ 12	3172	3.04 (3 to 3.09)	0.02	- 0.04 (- 0.11 to 0.03)	- 1.3	1.000
> 12 to ≤ 14	31,629	3.08 (3.07 to 3.1)	0.01			
> 14 to ≤ 16	28,740	3.09 (3.08 to 3.1)	0.01	0.01 (- 0.03 to 0.04)	0.3	1.000
> 16 to ≤ 18	3207	3.04 (3 to 3.08)	0.02	- 0.04 (- 0.12 to 0.03)	- 1.3	1.000
≤ 2	573	1.86 (1.78 to 1.93)	0.04	0.22 (0.1 to 0.34)	13.4	< 0.001
> 2 to ≤ 4	1272	1.87 (1.81 to 1.92)	0.03	0.23 (0.14 to 0.31)	14.0	< 0.001
> 4 to ≤ 6	979	1.86 (1.79 to 1.92)	0.03	0.23 (0.11 to 0.32)	14.0	< 0.001
**Triglyceride, mmol/L**						
> 6 to ≤ 8	564	1.68 (1.6 to 1.75)	0.04	0.04 (- 0.09 to 0.16)	2.4	1.000
> 8 to ≤ 10	1238	1.61 (1.56 to 1.66)	0.03	- 0.03 (- 0.12 to 0.06)	- 1.8	1.000
> 10 to ≤ 12	3172	1.64 (1.61 to 1.68)	0.02	0 (- 0.06 to 0.06)	0.0	1.000
> 12 to ≤ 14	31,629	1.64 (1.63 to 1.65)	0.01			
> 14 to ≤ 16	28,740	1.62 (1.61 to 1.63)	0.01	- 0.02 (- 0.05 to 0.01)	- 1.2	0.711
> 16 to ≤ 18	3207	1.63 (1.6 to 1.67)	0.02	0 (- 0.07 to 0.06)	0.0	1.000
≤ 2	573	4.79 (4.69 to 4.89)	0.05	- 0.25 (- 0.42 to - 0.09)	- 5.0	< 0.001
> 2 to ≤ 4	1272	4.78 (4.71 to 4.85)	0.04	- 0.26 (- 0.39 to - 0.14)	- 5.2	< 0.001
> 4 to ≤ 6	979	4.84 (4.75 to 4.92)	0.04	- 0.2 (- 0.35 to -0.06)	- 4.0	< 0.001
**Total cholesterol, mmol/L**						
> 6 to ≤ 8	564	4.83 (4.73 to 4.93)	0.05	- 0.21 (- 0.38 to - 0.04)	- 4.2	0.002
> 8 to ≤ 10	1238	5 (4.93 to 5.07)	0.04	- 0.04 (- 0.17 to 0.08)	- 0.8	1.000
> 10 to ≤ 12	3172	4.96 (4.91 to 5.01)	0.02	- 0.08 (- 0.16 to 0)	- 1.6	0.080
> 12 to ≤ 14	31,629	5.04 (5.03 to 5.06)	0.01			
> 14 to ≤ 16	28,740	5.04 (5.02 to 5.05)	0.01	0 (- 0.04 to 0.04)	0.0	1.000
> 16 to ≤ 18	3207	4.96 (4.91 to 5.01)	0.02	- 0.08 (- 0.17 to 0)	- 1.6	0.064
≤ 2	573	1.25 (1.22 to 1.28)	0.01	- 0.03 (- 0.08 to 0.01)	- 2.3	0.891
> 2 to ≤ 4	1272	1.23 (1.21 to 1.25)	0.01	- 0.05 (- 0.09 to - 0.02)	- 3.9	< 0.001
> 4 to ≤ 6	979	1.26 (1.23 to 1.28)	0.01	- 0.02 (- 0.07 to 0.02)	- 1.6	1.000
**HDL-C, mmol/L**						
> 6 to ≤ 8	564	1.24 (1.21 to 1.27)	0.01	- 0.04 (- 0.09 to 0.01)	- 3.1	0.191
> 8 to ≤ 10	1238	1.25 (1.23 to 1.27)	0.01	- 0.03 (- 0.06 to 0.01)	- 2.3	0.510
> 10 to ≤ 12	3172	1.24 (1.23 to 1.26)	0.01	- 0.04 (- 0.06 to - 0.01)	- 3.1	< 0.001
> 12 to ≤ 14	31,629	1.28 (1.28 to 1.29)	0			
> 14 to ≤ 16	28,740	1.28 (1.28 to 1.28)	0	0 (- 0.01 to 0.01)	0.0	1.000
> 16 to ≤ 18	3207	1.24 (1.22 to 1.25)	0.01	- 0.04 (- 0.07 to - 0.02)	- 3.1	< 0.001
≤ 2	573	3.54 (3.44 to 3.64)	0.05	- 0.22 (- 0.38 to - 0.06)	- 5.9	0.001
> 2 to ≤ 4	1272	3.55 (3.48 to 3.62)	0.04	- 0.21 (- 0.33 to - 0.09)	- 5.6	< 0.001
> 4 to ≤ 6	979	3.58 (3.5 to 3.67)	0.04	- 0.18 (- 0.32 to - 0.04)	- 4.8	0.002
> 6 to ≤ 8	564	3.59 (3.49 to 3.69)	0.05	- 0.17 (- 0.34 to 0)	- 4.5	0.038
> 8 to ≤ 10	1238	3.75 (3.67 to 3.82)	0.04	- 0.01 (- 0.14 to 0.11)	- 0.3	1.000
**Non-HDL-C, mmol/L**						
> 10 to ≤ 12	3172	3.72 (3.67 to 3.77)	0.02	- 0.04 (- 0.12 to 0.04)	- 1.1	1.000
> 12 to ≤ 14	31,629	3.76 (3.75 to 3.78)	0.01			
> 14 to ≤ 16	28,740	3.76 (3.74 to 3.77)	0.01	0 (- 0.04 to 0.04)	0.0	1.000
> 16 to ≤ 18	3207	3.72 (3.67 to 3.77)	0.02	- 0.04 (- 0.13 to 0.04)	- 1.1	1.000
LDL-C - low density lipoprotein cholesterol. HDL-C - high density lipoprotein cholesterol. The > 12 to ≤ 14-hour fasting group was used as the reference group, and the statistical evaluations were conducted by comparing other fasting groups to this group. P < 0.05 was considered statistically significant.						

**Figure 1:**
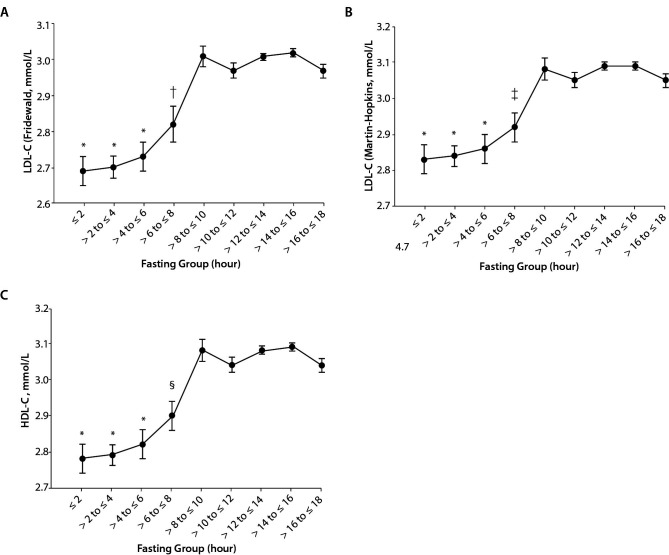
Low density lipoprotein cholesterol (LDL-C) concentrations estimated by the Friedewald (A), Martin-Hopkins (B), and Sampson/NIH (C) equations as a function of different fasting durations. Values are presented as marginal means with standard errors. Significant differences between the > 12 to ≤ 14-hour fasting group and other groups are indicated by * (P < 0.001), † (P = 0.002), ‡ (P = 0.007), and § (P = 0.003). HDL-C - high density lipoprotein cholesterol.

**Figure 2:**
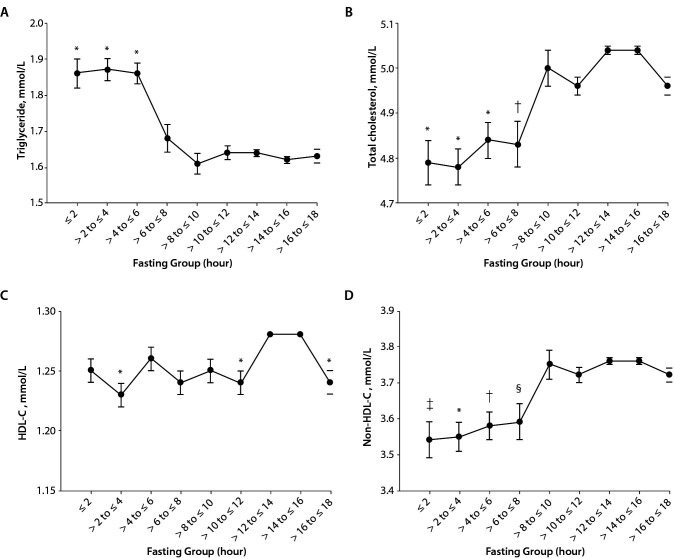
Triglyceride (A), total cholesterol (B), high density lipoprotein cholesterol (HDL-C) (C), and non-HDL-C (D) concentrations as a function of different fasting durations. Values are presented as marginal means with standard errors. Significant differences between the > 12 and ≤ 14-hour fasting groups are indicated by * (P < 0.001), † (P = 0.002), ‡ (P = 0.001), and § (P = 0.038).

Furthermore, the estimated marginal mean values with standard error for LDL-C, plotted based on the fasting groups, are illustrated in Figure 1, while those for triglycerides, total cholesterol, HDL-C, and non-HDL-C are shown in Figure 2.

Concentrations of LDL-C, regardless of the three calculation methods, were statistically significantly lower in the ≤ 2 (P < 0.001), > 2 to ≤ 4 (P < 0.001), > 4 to ≤ 6 (P < 0.001), and > 6 to ≤ 8 (Friedewald: P = 0.002, Sampson-NIH: P = 0.003, Martin-Hopkins: P = 0.007) fasting groups compared to > 12 to ≤ 14 hours. The absolute mean difference was decreased as the fasting duration increased, and beginning from the > 8 to ≤ 10 hours fasting group, this difference ranged between 0 and - 0.04. The highest mean difference was observed in LDL-C estimation using the Friedewald equation, with a value of - 0.32 mmol/L (- 10.6%), followed by the Sampson-NIH equation at - 0.30 mmol/L (- 9.7%), and the Martin-Hopkins equation at - 0.26 mmol/L (- 8.4%).

Similarly, statistically significant lower concentrations of total cholesterol were observed in the ≤ 2 (P < 0.001), > 2 to ≤ 4 (P < 0.001), > 4 to ≤ 6 (P < 0.001), and > 6 to ≤ 8 (P = 0.002) fasting groups compared to the > 12 to ≤ 14 hour fasting group. The greatest mean difference was recorded at - 0.26 mmol/L (- 5.2%).

A significant negative bias in HDL-C mean values was observed between the > 12 to ≤ 14 hour fasting group and the > 2 to ≤ 4 (P < 0.001), > 10 to ≤ 12 (P < 0.001), and > 16 to ≤ 18 (P < 0.001) fasting groups. However, these differences were relatively modest, with the greatest being - 0.05 mmol/L.

For non-HDL-C concentrations, a significant negative bias was also observed between the > 12 to ≤ 14 hour fasting group and the ≤ 2 (P = 0.001), > 2 to ≤ 4 (P < 0.001), > 4 to ≤ 6 (P = 0.002), and > 6 to ≤ 8 (P = 0.038) fasting groups. The greatest mean difference observed was - 0.22 mmol/L (- 5.9%).

Conversely, for triglycerides, statistically significant higher concentrations were found in the ≤ 2 (P < 0.001), > 2 to ≤ 4 (P < 0.001), and > 4 to ≤ 6 (P < 0.001) fasting groups compared to the > 12 to ≤ 14 hour fasting group, with the highest mean difference documented at 0.23 mmol/L (14%).

When comparing the percentage differences in LDL-C concentrations estimated by the three equations with biological variation values, the differences exceeded the biological variation values in the ≤ 2, > 2 to ≤ 4, and > 4 to ≤ 6 hour fasting groups for the Friedewald and Sampson/NIH equations (*17*). For the Martin-Hopkins equation, only the ≤ 2 and > 2 to ≤ 4 hour fasting groups exceeded the biological variation value. However, none of the fasting groups surpassed the corresponding within-subject biological variation values for triglycerides, total cholesterol, HDL-C, and non-HDL-C.

## Discussion

This study found that LDL-C concentrations varied with fasting duration, regardless of the calculation method, up to at least 8 hours of fasting. The differences in LDL-C concentrations between fasting and nonfasting states were similar across the three calculation methods, with the greatest absolute mean differences being - 0.32, - 0.30, and - 0.26 mmol/L for the Friedewald, Sampson/NIH, and Martin-Hopkins equations, respectively.

Triglycerides were the component in calculating LDL-C that exhibited the most pronounced variation with fasting duration. Fluctuations in triglyceride concentrations persisted for at least 6 hours of fasting, with a highest observed variation of 14%. In contrast, HDL-C concentrations showed no substantial variation with fasting duration, while both total cholesterol and non-HDL-C concentrations decreased significantly after at least 8 hours of fasting.

Previous population-based studies have examined the influence of habitual food intake on the plasma lipid profile (*18*-*21*). These studies evaluated only LDL-C concentrations calculated using the Friedewald equation, as the Martin-Hopkins and Sampson/NIH equations were not yet developed at that time. To the best of our knowledge, this study is the first to provide data on the variation of LDL-C concentrations estimated by the Martin-Hopkins and Sampson/NIH equations based on fasting duration.

In their investigation of 33,391 individuals in the Copenhagen General Population Study from Denmark, Langsted *et al.* reported a maximum mean change from fasting concentrations of - 0.2 mmol/L for LDL-C and total cholesterol, - 0.1 mmol/L for HDL-C, and + 0.3 mmol/L for triglycerides (*18*). The changes have been considered clinically unimportant. The decrease in LDL-C and total cholesterol concentrations has been explained by fluid intake together with food intake, thus resulting in hemodilution (*18*).

In a more recent report, the findings from the cited study were reevaluated by integrating data from another study involving the Danish general population (*18*, *19*). Based on data from 92,285 individuals, similar maximal mean changes in related lipid parameters after habitual food consumption have been noted. These changes have also continued to be considered clinically insignificant (*19*).

In a community-based population study involving 209,180 individuals from the Calgary Laboratory Service in Canada, Sidhu and Naugler examined the association between fasting duration and standard lipid profile results (*20*). They found variations of up to 10% for LDL-C and 20% for triglycerides, with no significant changes noted for HDL-C and total cholesterol. The authors concluded that fasting is largely unnecessary for routine lipid concentrations assessments.

In another prospective study of 26,330 healthy women from the Women’s Health Study in the USA, Mora *et al.* reported modest decreases of 1% to 5% in nonfasting LDL-C, total cholesterol, and non-HDL-C concentrations compared to fasting concentrations, with no difference observed for HDL-C (*21*). They also noted that triglyceride concentrations were 15% higher in the nonfasting state. The authors considered these changes clinically insignificant, except for the increase in triglyceride concentrations.

Distinct differences exist in the frequency and timing of meals, as well as the proportion of daily energy intake *per* meal, across various cultures and populations (*22*, *23*). Consequently, it might be anticipated that postprandial variations in lipid concentrations would differ from one country to another. However, the degree of lipid concentration alteration based on fasting durations has been generally consistent across studies conducted in countries such as Denmark, Canada, USA, and Türkiye, where our study was conducted.

In the previously cited studies, the degree of change in lipid concentrations based on fasting status has been described as “clinically insignificant” or “clinically unimportant”. Nevertheless, these studies have not specified the criteria used to reach such conclusions (*18*-*21*).

On the other hand, Cartier *et al.* measured lipid parameters in the same individuals under both fasting and nonfasting states, assessing the difference between the two states based on biological variation observed during fasting (*24*). The authors attributed a substantial portion of the observed changes in total cholesterol, HDL-C, and non-HDL-C to biological variation. However, they noted that postprandial changes in LDL-C and triglyceride concentrations exceeded biological variation in a significant number of cases.

In the present study, when comparing variations in lipid concentrations based on fasting durations with the within-subject biological variation values obtained from the fasting state, it was determined that the differences observed for LDL-C, calculated using the Friedewald and Sampson/NIH equations up to at least 6 hours postprandial, and for LDL-C calculated using the Martin-Hopkins equation up to at least 4 hours postprandial, exceeded the within-subject biological variation values obtained from the EFLM BV database. However, the postprandial variations observed in triglycerides, total cholesterol, and HDL-C remained below the biological variation across all fasting durations.

We also assessed postprandial lipid fluctuations following the recommendations of the 2019 ESC/EAS Guidelines for managing dyslipidemia (*3*). In this guideline, total cholesterol is utilized alongside several other risk factors to estimate the total cardiovascular risk using the SCORE system. The risk scoring is sensitive to changes in total cholesterol concentrations, with a 1 mmol/L alteration potentially influencing the estimated risk. However, the observed decrease of 0.26 mmol/L in total cholesterol concentrations in our study is unlikely to have a significant impact on the calculated 10-year cardiovascular risk.

The determination of intervention strategies, including decisions on drug therapy indications, is based on both the total cardiovascular risk and untreated LDL-C concentrations (*3*). There are six categories based on LDL-C concentrations, with two representing exact values and four representing specific ranges. The interval widths for these ranges are 0.4 mmol/L in two categories and 0.8 and 1.9 mmol/L in the others. As a result, a postprandial decrease of 0.3 mmol/L in LDL-C concentrations could potentially shift an individual into a different category, particularly in those with an interval width of 0.4 mmol/L. This likelihood diminishes when postprandial reductions fall below 0.1 mmol/L. In this study, postprandial changes of less than 0.1 mmol/L were observed across all three equations for fasting durations of at least 8 hours. It should also be noted that not all alterations in categories result in a change in the intervention strategy. Therefore, if a blood sample is collected following a fasting period of less than 8 hours and the LDL-C concentration is near the boundary of a category where a shift could alter the treatment approach, caution should be exercised.

In addition to determining drug therapy indications, LDL-C is also crucial for setting treatment targets and goals in the prevention of cardiovascular disease. For individuals at very high or high cardiovascular risk, a key therapeutic objective is to achieve at least a 50% reduction in LDL-C from baseline concentrations (*3*). The term ‘baseline’ refers to the LDL-C concentration in individuals who are not taking lipid-lowering medications (*3*). Therefore, when baseline or post-treatment LDL-C measurements are taken with varying fasting durations, differences of up to 0.3 mmol/L may occur, potentially leading to inaccurate percentage change calculations and erroneous evaluations. Based on the data from our study, it seems that a fasting period of at least 8 hours is required across all three LDL-C calculation methods to accurately assess the reduction in LDL-C concentrations following lipid-lowering therapy.

There are also specific treatment goals for LDL-C across different cardiovascular risk categories, including concentrations such as 1.0, 1.4, 1.8, 2.6, and 3.0 mmol/L (*3*). When an LDL-C result is close to a treatment decision threshold, it is crucial to consider the potential impact of postprandial variations on determining whether the treatment has successfully achieved the desired target.

Lipid testing in a nonfasting state presents numerous practical benefits for patients, laboratories, and physicians (*10*). Eliminating the need for patients to return for fasting tests improves compliance and decreases the likelihood of missing important lipid assessments. The benefit to laboratories is that the workload, usually concentrated in the morning, is spread throughout the day. Clinicians can promptly review and make decisions regarding lipid profiles, eliminating the additional follow-up communications or visits (*10*, *19*, *25*). Moreover, beyond these practical advantages, nonfasting lipid profiles are suggested to offer a more accurate estimation of cardiovascular disease risk compared to fasting lipids (*10*, *19*, *26*, *27*). This is attributed to their ability to capture the average atherogenic lipoprotein load over a 24-hour period, reflecting the fact that in real life, we spend the majority of our time in a postprandial state (*19*, *26*, *27*). Finally, nonfasting lipid testing has also the potential to reduce the risk of hypoglycemia in fasting diabetic patients (*19*).

Currently, the EAS and EFLM consensus-based recommendations state that fasting is generally not required for lipid testing (*2*). However, fasting is advised when nonfasting triglycerides are ≥ 4.5 mmol/L, and it is recommended for certain clinical conditions associated with hypertriglyceridemia, as well as when additional tests requiring fasting are necessary (*2*). Additionally, the consensus paper suggests that if test results are close to therapeutic decision thresholds, they should ideally be confirmed by at least two repeated measurements using the same method, with the results averaged. This recommendation stems from the risk of analytical errors influencing clinical decisions (*2*). However, the paper does not specify the fasting conditions under which additional lipid measurements should be conducted. Based on the findings of this study, we stress that, in addition to addressing analytical variation, further lipid measurements should be performed in a fasting state to minimize the impact of postprandial variation on lipid results.

Direct measurement of LDL-C concentration can be performed using automated homogeneous enzyme assays developed by various manufacturers (*28*). Rather than performing direct LDL-C measurements in all patients, recent guidelines recommend direct measurement of LDL-C when triglyceride concentrations exceed 4.5 mmol/L or when LDL-C concentrations are below 1.3 mmol/L or 1.8 mmol/L (*1*-*4*). Direct LDL-C results should theoretically remain stable regardless of fasting status, as these assays are minimally affected by chylomicrons and chylomicron remnants (*29*). Consequently, direct measurement of LDL-C may be considered as an alternative when calculated LDL-C concentrations in a nonfasting state are near clinical decision thresholds, thereby eliminating the necessity of collecting a new sample in a fasting state. In the current study, we were unable to evaluate postprandial direct LDL-C variations due to the unavailability of direct LDL-C results. Therefore, further studies are needed to assess the impact of different fasting durations on directly measured LDL-C concentrations. Additionally, it is important to consider the limitations of direct LDL-C assays, including variability between assay methods and potential inaccuracies in certain dyslipidemic samples (*2*, *3*, *28*).

In addition to the lack of direct LDL-C measurement, the present study has several limitations. First, lipid measurements were not obtained from the same individuals at different fasting durations. Second, the time since the last meal was self-reported, introducing the possibility of recall bias. Third, although the data analysis adjusted for factors influencing lipid concentrations, such as age, gender, and the time of day of blood collection, other relevant factors such as diabetes and lipid-lowering drug treatment were not available for inclusion in the analysis. However, a previous study reported that the findings remained unchanged when the dataset was adjusted for lipid-lowering therapy, in addition to age and gender (*18*). Conversely, the authors observed a decrease in LDL-C concentration of 0.6 mmol/L within 1-3 hours after a meal in diabetic patients, nearly double the magnitude of change observed for LDL-C in our study.

In conclusion, LDL-C concentrations estimated by the Friedewald, Sampson/NIH, and Martin-Hopkins equations, regardless of the calculation method, significantly decreased up to 8 to 10 hours postprandially compared to the fasting state (defined as 12 to 14 hours). Among the components of these equations, triglyceride concentrations were the most sensitive to fasting duration, remaining significantly elevated for 4 to 6 hours postprandially, while total cholesterol and non-HDL-C concentrations decreased up to 8 to 10 hours. However, HDL-C concentrations remained relatively stable. Therefore, if nonfasting LDL-C concentrations are near clinical decision thresholds, additional measurements should be taken after at least 8 hours of fasting to ensure patient safety.

## Data Availability

The data generated and analyzed in the presented study are available from the corresponding author on request.
